# Identification and validation of diagnostic genes associated with neutrophil extracellular traps of type 2 diabetes mellitus

**DOI:** 10.3389/fgene.2024.1373807

**Published:** 2024-09-04

**Authors:** Meifang He, Jin Niu, Haihua Cheng, Chaoying Guo

**Affiliations:** Endocrinoloy Department, Peking University First Hospital Taiyuan Hospital (Taiyuan Central Hospital), Taiyuan, China

**Keywords:** neutrophil extracellular traps, type 2 diabetes mellitus, GEO, bioinformatic, diagnosis

## Abstract

**Background:**

Neutrophil extracellular traps (NETs) cause delayed wound closed up in type 2 diabetes mellitus (T2DM), but the specific regulatory mechanism of NETs-related genes (NETs-RGs) in T2DM is unclear.

**Methods:**

We acquired GSE21321 and GSE15932 datasets from gene expression omnibus (GEO) database. First, differentially expressed genes (DEGs) between T2DM and control samples of GSE21321 dataset were sifted out by differential expression analysis. NETs scores were calculated for all samples in GSE21321 dataset, and key module genes associated with NETs scores were screened by constructing co-expression network. Then, DEGs and key module genes were intersected to yield intersection genes, and candidate genes were identified by constructing a protein protein interaction (PPI) network. Least absolute shrinkage and selection operator (LASSO) regression analysis was implemented on candidate genes to screen out diagnostic genes, and they were subjected to single sample gene set enrichment analysis (ssGSEA). Finally, immune characteristic analysis was carried out, and we constructed the gene-drug and transcription factor (TF)-miRNA-mRNA networks. Besides, we validated the expression of diagnostic genes by quantitative real-time polymerase chain reaction (qRT-PCR).

**Results:**

In total, 23 candidate genes were gained by PPI analysis. The 5 diagnostic genes, namely, inter-trypsin inhibitor heavy chain 3 (ITIH3), fibroblast growth factor 1 (FGF1), neuron cell adhesion molecule (NRCAM), advanced glycosylation end-product-specific receptor (AGER), and calcium voltage-gated channel subunit alpha1 C (CACNA1C), were identified via LASSO analysis, and they were involved in carboxylic acid transport, axonogenesis, etc. M2 Macrophage, Monocyte, Natural killer (NK) cell, and Myeloid dendritic cells (DC) were remarkably different between T2DM and control samples. Diagnostic genes had the strongest and the most significant positive correlation with B cells. The gene-drug network included CACNA1C-Isradipine, CACNA1C-Benidipine and other relationship pairs. Totally 76 nodes and 44 edges constituted the TF-miRNA-mRNA network, including signal transducer and activator of transcription 1(STAT1) -hsa-miR-3170-AGER, CCCTC-binding factor (CTCF)-hsa-miR-455-5p-CACNA1C, etc. Moreover, qRT-PCR suggested that the expression trends of FGF1 and AGER were in keeping with the results of bioinformatic analysis. FGF1 and AGER were markedly regulated downwards in the T2DM group.

**Conclusion:**

Through bioinformatic analysis, we identified NETs-related diagnostic genes (ITIH3, FGF1, NRCAM, AGER, CACNA1C) in T2DM, and explored their mechanism of action from different aspects, providing new ideas for the studies related to diagnosis and treatment of T2DM.

## 1 Introduction

With the progress of society, the incidence and prevalence of type 2 diabetes mellitus (T2DM) has sharply risen due to the change of lifestyle, environmental pollution, mental stress and other factors, and has become a serious global health problem. T2DM is caused by a combination of genetic and environmental factors that affect the metabolism of sugar, fat and protein. Extended lasting hyperglycemia can cause multi-system harm to organs and tissues, such as, eyes (Tan and Wong, 2023), kidneys (Thipsawat, 2021), nerves (Yang et al., 2022), heart and blood vessels (Marassi and Fadini, 2023). The pathogenesis of T2DM is complex. There is research indicating a certain correlation between T2DM and Alzheimer’s disease (Surguchov, 2020; Caveolin et al., 2020). We know that there are many tissues and organs, such as pancreas, liver, muscle, kidney, heart, brain and adipose tissue, are involved in regulating blood glucose metabolism. Although there are many clinical hypoglycemic drugs that can bring many benefits to patients, they still cannot prevent the emergence of serious complications of T2DM, such as diabetic foot, diabetic nephropathy, coronary heart disease, etc., which not only cause patients great physical and psychological pain, but also a heavy financial burden. Many studies have shown that T2DM is also considered an autoimmune disease (Itariu and Stulnig, 2014; Wang et al., 2023; Zhou et al., 2018). Including congenital immune response deficits (neutrophils and macrophages dysfunction) and adaptive immune response dysfunction (T cells) (Berbudi et al., 2020). Chronic hyperglycemia increases levels of various chemokines like monocyte chemokine protein-1 (MCP-1) and pro-inflammatory cytokines such as Tumour Necrosis Factor alpha (TNF-α), interleukin-1beta (IL-1β), and interleukin-6(IL-6) (Amorim et al., 2023). These inflammatory factors can infiltrate macrophages in the pancreas and adipose tissue, leading to insulin resistance and islet cell dysfunction (Jo and Fang, 2021).

Neutrophil extracellular traps (NETs) are made up of filaments of chromatin DNA coated with granular proteins that can be released by neutrophils to trap microorganisms (Brinkmann and Zychlinsky, 2007; Yang et al., 2020). NETs are part of the innate immune response, which is beneficial to the human body, but in some cases, the disorder of NETs can also cause pathological effects on the human body (Yang et al., 2020; Papayannopoulos et al., 2010). The formation of NETs is due to the induction of NETosis (inflammatory cell death mode of neutrophils) under the stimulation of various pathogens, activated platelets, chemokines, phorbol esters, etc., directly activating the protein kinase C (PKC) and Raf-MEK-ERK-MAP kinase pathways (Reeves et al., 2002; Smith et al., 2014). The activation of MAP kinase will initiate the formation of NADPH oxidase complex, rapidly generating reactive oxygen species (ROS). Meanwhile, key proteins in NETosis, neutrophil elastase (NE) and myeloperoxidase (MPO) (Parker and Winterbourn, 2012; Hawez et al., 2019), contribute to nuclear membrane permeability and further development of chromatin; Peptidylarginine deiminase 4 (PAD4) modifies histones by converting arginine to citrulline, leading to chromatin depolymerization (Lewis et al., 2015; Wang et al., 2009). Chromatin is released outside the cell through membrane pores, ultimately releasing DNA, citrulline histone (citH3), and other intracellular particles to form a cell capture network. Owing to the unspecific action of the emitted enzyme proteins, NETs may bring out an unregulated inflammatory response leading to pathological changes resulting in direct cell damage. NETs also recruit extra pro-inflammatory cytokines, inducing the production of autoantibodies, forming immune complexes, and causing tissue damage (Petretto et al., 2019; Bruschi et al., 2020). NETs have been demonstrated to join in the pathophysiological processes of various diseases, for instance autoimmune diseases, cancer, chronic inflammation, delayed wound healing (Liu et al., 2019; Hirota et al., 2020), etc. In recent years, relevant literature has reported that NETs are related to the occurrence and development of diabetic nephropathy, diabetic foot ulcers and diabetes-related atherosclerotic diseases in diabetic cases (Xu et al., 2019; Borissoff et al., 2013).However, the definite regulatory mechanism of NETs-related genes (NETs-RGs) in T2DM remains indefinite.

In this study the relevant data sets of T2DM, including GSE21321 and GSE15932, was obtained from the Gene Expression Omnibus (GEO) database. NETs-RGs were drawn out from the literature (Wu et al., 2022). Then the diagnostic genes were screened through bioinformatic analyses such as differential expression analysis and weighted gene co-expression network analysis (WGCNA) and least absolute shrinkage and selection operator (LASSO) analysis. Moreover, the gene set enrichment analysis (GSEA), immune profile analysis, construction of the gene-drug network and transcription factor (TF)-miRNA-mRNA network were also performed on diagnostic genes to explore their mechanism of action in T2DM.This research is of great significance for the diagnosis and treatment of T2DM.

## 2 Materials and methods

### 2.1 Sources of data

T2DM-related datasets, microarray and RNA-seq of GSE21321 and GSE15932 were acquired from the GEO database (https://www.ncbi.nlm.nih.gov/geo/). Among them, GSE21321 contained blood samples from 9 T2DM and 8 control, and GSE15932 contained peripheral blood samples from 8 T2DM and 8 control. The platform of GSE21321 was GPL6883 Illumina HumanRef-8 v3.0 expression beadchip, and GSE21321 was applied as training set. The platform of GSE15932 was GPL570 [HG-U133 Plus 2] Affymetrix Human Genome U133 Plus 2.0 Array, and GSE15932 was employed as validation set to verify the expression of the diagnostic genes and the diagnostic performance for the disease (Table 1). The 137 NETs-RGs were extracted from the literature (Supplementary Table S1) (Wu et al., 2022).

**TABLE 1:** Information on the datasets used in this study.

Dataset	T2DM	Control	Platform
GSE21321	9	8	GPL6883
GSE15932	8	8	GPL570

### 2.2 Differential expression analysis and WGCNA

In the GSE21321 dataset, the differentially expressed genes (DEGs) between T2DM and control samples were sifted out by limma package (version 3.46.0) (Wang et al., 2021) setting *p* < 0.05 and |log_2_FC| > 0.5. Next, NETs scores were calculated for all samples in GSE21321 dataset utilizing the GSVA package (version 1.38.2) (Su et al., 2022), and the difference of NETs score between T2DM and control samples was compared by rank sum test. Then, to screen for key module genes associated with NETs scores, we constructed a co-expression network using WGCNA (Langfelder and Horvath, 2008). WGCNA serves as an algorithmic approach tailored for scrutinizing gene expression patterns across a multitude of samples. It possesses the ability to cluster genes and erect modules predicated on akin gene expression patterns, all the while scrutinizing the correlations between said modules and biological traits. Firstly, the samples in the training set were clustered and outliers were eliminated, then this network was constructed based on a soft threshold of *R* ^2^ over 0.85 and connectivity close to 0. The adjacency matrix was then converted into the topological overlap matrix (TOM). Subsequently, the similar genes were classified into different modules based on dynamic tree cutting, and different colors were employed to express different modules. The NETs score was treated as the trait, Preason was then utilized to explore the correlation between these modules and the traits (*p* < 0.05), and the most remarkably correlated module was selected as the key module, and then the genes therein were filtered according to |gene significance (GS)| > 0.6 and |module membership (MM)| > 0.8) as key module genes.

### 2.3 Functional enrichment analysis of intersected genes and framework of a PPI network

We identified the intersection genes by intersecting differentially expressed genes (DEGs) with key module genes. Subsequently, the intersection genes were enrichment analysed using clusterProfiler (version 4.4.4) (Yu et al., 2012) and org.Hs.eg.db (version 3.12.0) (Lowe et al., 1986) packages to explore the pathways and functions they were related to, encompassing gene ontology (GO) and kyoto encyclopedia of genes and genomes (KEGG) (adj.*p* < 0.05). To further understand the interaction relationships among intersection genes, PPI network (Confidence = 0.4, the discrete proteins were removed) of intersecting genes was constructed in the STRING database (https://string-db.org) and set degree cutoff = 2, node score cutoff = 0.2, k-core = 0.6, max. depth = 100 to screen candidate genes.

### 2.4 Acquisition of diagnostic genes

First, in the GSE21321 dataset, LASSO analysis was implemented on candidate genes to screen out diagnostic genes, and we compared the expressions of diagnostic genes between T2DM and control samples in training set and validation set. Then, receiver operating characteristic (ROC) curves were plotted utilizing the pROC package (version 1.17.0.1) (Robin et al., 2011), and area under the curve (AUC) values were calculated to assess the diagnostic ability of diagnostic genes for T2DM. Further, the nomogram containing diagnostic genes was constructed using rms package (version 6.2–0) (Liu T. T. et al., 2021) to predict risk rates for T2DM, and its predictive value was assessed by ROC curve. Similarly, the diagnostic performance of diagnostic genes for T2DM was verified in the validation set. To probe the relevant pathways involved in diagnostic genes, we conducted gene set enrichment analysis (GSEA) on them by clusterProfiler package (version 3.18.0) (Yu et al., 2012) based on the GO-Biological process (BP) database.

### 2.5 Immune characteristic analysis

The study calculated the 10 immune cell proportions (scores) for all samples utilizing the quantiseq algorithm of immunedeconv (version 2.0.4). After that, the immune cell proportions were compared between T2DM and control samples by rank sum test. Eventually, spearman correlation analysis between among immune cells, and diagnostic genes and immune cells were carried out.

### 2.6 Framework of gene-drug and TF-miRNA-mRNA networks

In order to identify small-molecule compounds with potential therapeutic effects on T2DM, we predicted the target drugs associated with diagnostic genes via DrugBank database (https://go.drugbank.com/), and constructed gene-drug network by Cytoscape software. Then, TFs and target miRNAs of diagnostic genes were predicted via NetworkAnalyst (https://www.networkanalyst.ca/) and miRWalk (http://mirwalk.umm.uni-heidelberg.de/) databases, respectively, the TF-mRNA and miRNA-mRNA relationship pairs were merged to yield the TF-miRNA-mRNA regulatory network.

### 2.7 Quantitative real-time polymerase chain reaction (qRT-PCR)

We collected fresh blood samples of T2DM (n = 10) and control (n = 10) groups from the central hospital of Taiyuan city. All participants have signed informed consent forms and have been agreed by the hospital ethics committee. The characteristic information of these patients is in Supplementary Table S2. We extracted 500 μL of frozen blood from the samples and added TRIzol reagent to extract total RNA. Afterwards, 1 μL of RNA was taken and the concentration was measured using a nanophotometer N50. Reverse transcription of mRNA was performed using Servicebio’s Surscript First Strand cDNA Synthesis Kit. Dilute the reverse transcription product cDNA with ddH2O without RNase/DNase and perform qPCR reaction by diluting it to 5–20 times. GAPDH serves as an internal reference. Eventually, compare the expression of diagnostic genes in T2DM and normal blood. The primer sequence is shown in Supplementary Table S3.

### 2.8 Statistical analysis

In this study all bioinformatics analyses were conducted using the R package. Differential expression analyses between T2DM and controls were executed via the limma package. Co-expression network construction utilised the WGCNA package. Enrichment analyses were performed in clusterProfiler and org.Hs.eg.db packages. ROC was plotted through pROC package. Nomograms were constructed utilising the rms package. Immunological analysis was implemented in immunedeconv. Differences between groups were compared using wilcoxon test. If not otherwise specified, *p* < 0.05 indicates statistical significance.

## 3 Results

### 3.1 Identification of DEGs and key module

**FIGURE 1:**
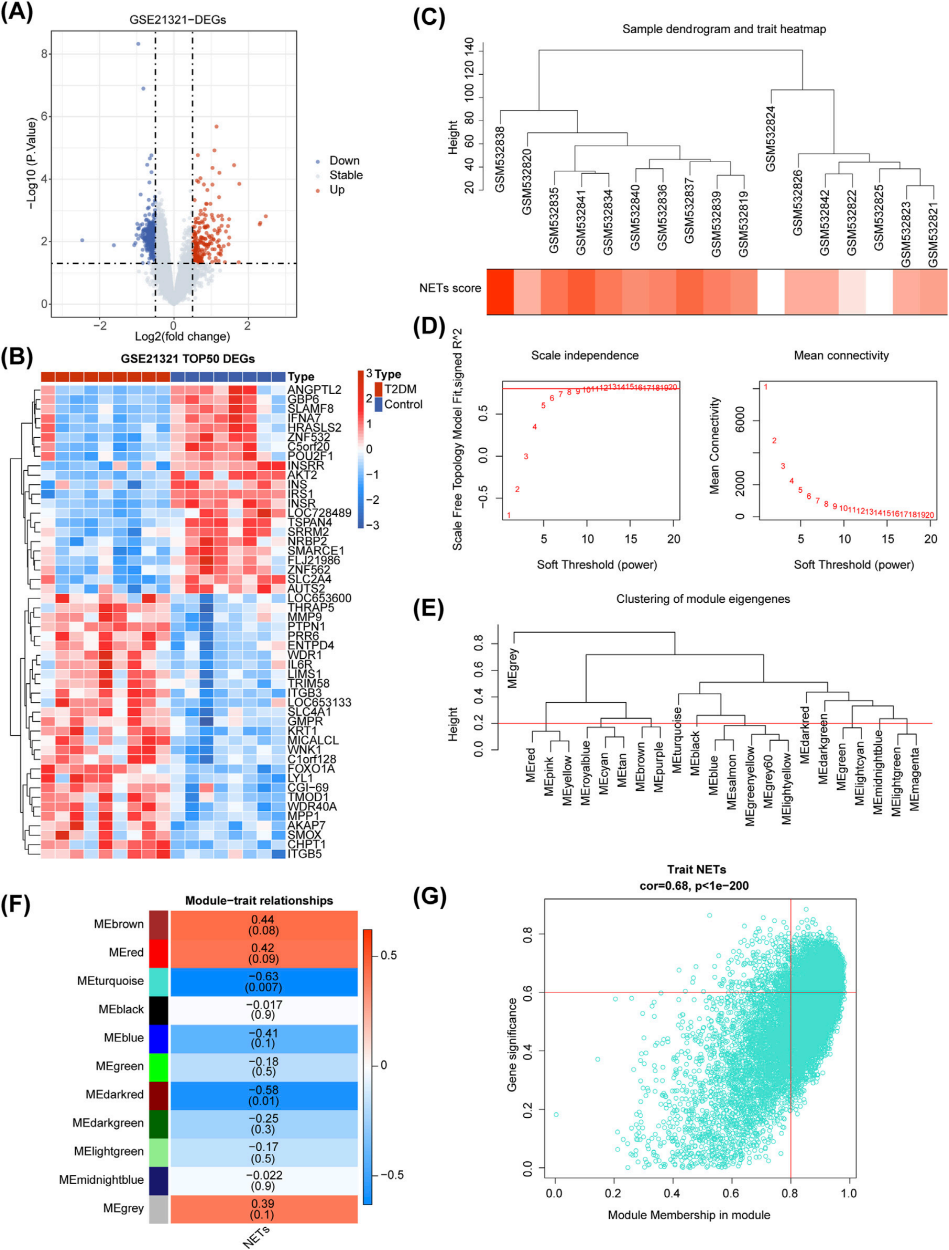
Differential expression analysis and Weighted Gene Co-expression Network Analysis (WGCNA) in the GSE21321 dataset. **(A, B)** The volcano map **(A)** and heatmap **(B)** of differentially expressed genes (DEGs) between type 2 diabetes mellitus (T2DM) and control samples. **(C**) Sample clustering and phenotypic heatmap. **(D)** Selection of the optimal soft-thresholding (power). **(E)** Module clustering tree. **(F)** Heatmap of the relationships between gene modules and NETs score. **(G)** Correlation between MEturquoise module gene and NETs scoring traits.

There were 671 DEGs between T2DM and control samples (Figure 1A), and the heat map illustrated the top50 of them (Figure 1B). As seen from the violin plot, the NETs score was remarkably higher in T2DM samples than that in the control samples (Supplementary Figure S1). In Figure 1C, it's evident that the clustering performance of the dataset samples was remarkably strong, indicating no necessity for sample exclusion. A soft threshold of 11 was applied to construct the scale-free network when R2 was approximately 0.85 and the connectivity was close to 0 (Figure 1D). Subsequently, genes were grouped into 11 modules (Figure 1E). Among which, MEturquoise (Cor = −0.63 and *p* < 0.05) had the strongest correlation with NETs score, so we treated it as the key module (Figure 1F). MEturquoise module contained 3,368 key module genes (|GS| > 0.6 and |MM| > 0.8), and they were positively correlated with the NETs score (Figure 1G).

### 3.2 Functional enrichment and PPI analyses of intersection genes

**FIGURE 2:**
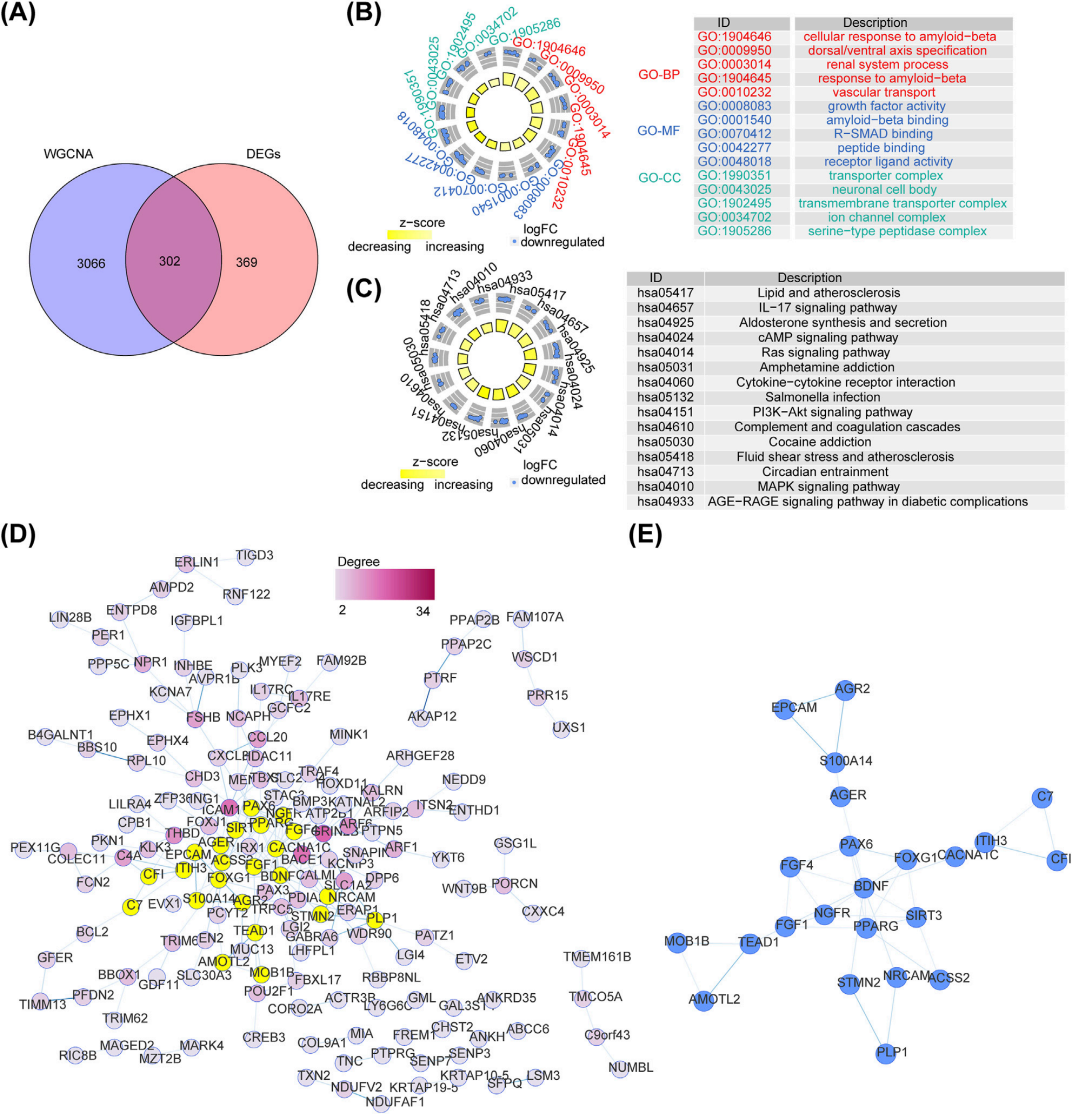
Identification of intersected genes and functional enrichment analysis. **(A)** The venn diagram of intersection gene between DEGs and key module genes in WGCNA. **(B)** The Gene Ontology (GO) terms enriched in intersected genes. **(C)** The Kyoto Encyclopedia of Genes and Genomes (KEGG) pathways enriched in intersected genes. **(D)** The Protein Protein interaction (PPI) network of intersected genes between DEGs and key module genes. **(E)** The interaction among 23 candidate genes.

DEGs and key module genes were intersected to yield 302 intersection genes (Figure 2A). GO items displayed that these genes were enriched to cellular response to amyloid−beta, growth factor activity, transporter complex and so on (Figure 2B). KEGG pathways revealed that intersection genes were primarily related to lipid and atherosclerosis, aldosterone synthesis and secretion, Interleukin 17(IL−17) signaling pathway, etc. (Figure 2C). A total of 165 nodes and 203 edges constituted the PPI network, containing Interleukin 17 Receptor E (IL17RE)-FAM92B, Brain-derived neurotrophic factor (BDNF)- calcium voltage-gated channel subunit alpha1 C (CACNA1C), peroxisome proliferator-activated receptor gamma (PPARG)- Paired box protein 6(PAX6) and other reciprocal relationship pairs (Figure 2D). After analyzing the topological properties of the PPI network, we acquired 23 candidate genes, such as PPARG, acetyl-CoA synthetase 2 (ACSS2), Complement factor I (CFI) (Figure 2E; Supplementary Table S4).

### 3.3 Excellent diagnostic power of diagnostic genes for T2DM

**FIGURE 3:**
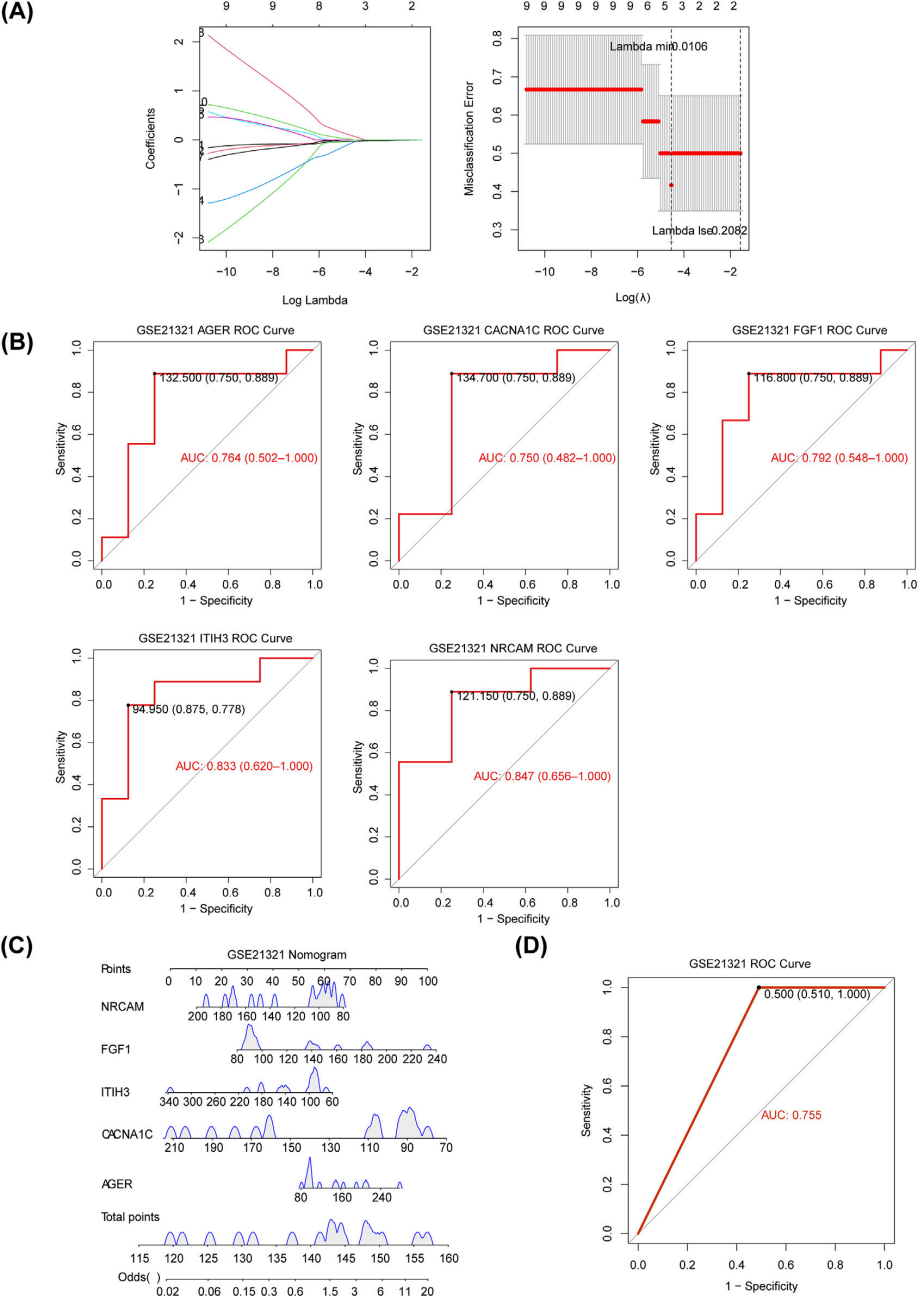
Identification of diagnostic genes for T2DM. **(A)** The plot of gene coefficients and error plots for 10-fold cross-validation in least absolute shrinkage and selection operator (LASSO) analysis. **(B)** The receiver operating characteristic (ROC) curves of diagnostic genes. AUC: area under the curve. **(C)** Construction of the nomogram based on the diagnostic genes in the GSE21321 dataset. **(D)** The ROC curve of the nomogram.

The five diagnostic genes, namely, inter-trypsin inhibitor heavy chain 3 (ITIH3), fibroblast growth factor 1 (FGF1), neuron cell adhesion molecule (NRCAM), advanced glycosylation end-product-specific receptor (AGER), and CACNA1C, were identified when the lambda min = 0.0106 via LASSO analysis (Figure 3A), and they were all downregulated in T2DM samples of the GSE21321 dataset (Supplementary Figure S2). Further,AUC values of ITIH3 (AUC = 0.833), FGF1 (AUC = 0.792), NRCAM (AUC = 0.847), AGER (AUC = 0.764), and CACNA1C (AUC = 0.750) were all greater than 0.7, indicating that diagnostic genes had strong diagnostic power for T2DM (Figure 3B). The nomogram suggested that the diagnostic genes had strong risk prediction ability for T2DM, and the results were further validated by the ROC curve (AUC > 0.7) (Figures 3C,D). Similarly, the diagnostic efficacy of diagnostic genes for T2DM was validated in the validation set (Supplementary Figures S3, S4).

### 3.4 Diagnostic genes were involved in multiple pathways

**FIGURE 4:**
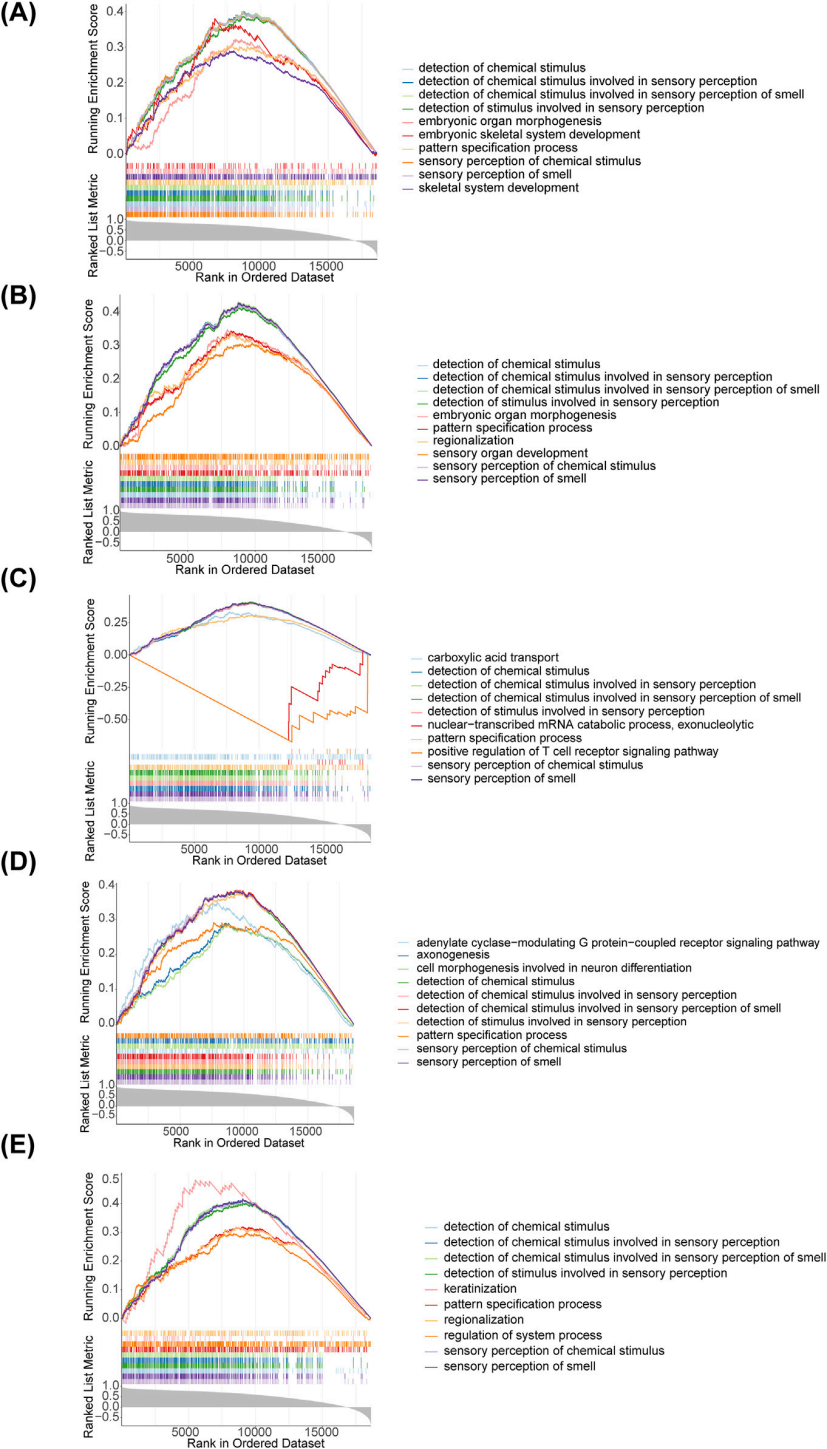
Gene Set Enrichment Analysis (GSEA) enrichment analysis of diagnostic genes. **(A)** AGER; **(B)** CACNA1C; **(C)** FGF1; **(D)** ITIH3; **(E)** NRCAM.

We applied GSEA to further explore the biological pathways that diagnostic genes were involved in T2DM, and found that all five diagnostic genes were mainly associated with detection of chemical stimulus, detection of chemical stimulus involved in sensory perception, detection of chemical stimulus involved in sensory perception of smell, detection of stimulus involved in sensory perception, pattern specification process, sensory perception of chemical stimulus and sensory perception of smell (Figure 4). This suggests that these genes may influence the onset and development of T2DM through multiple pathways.

### 3.5 Immuno-infiltration analysis

**FIGURE 5:**
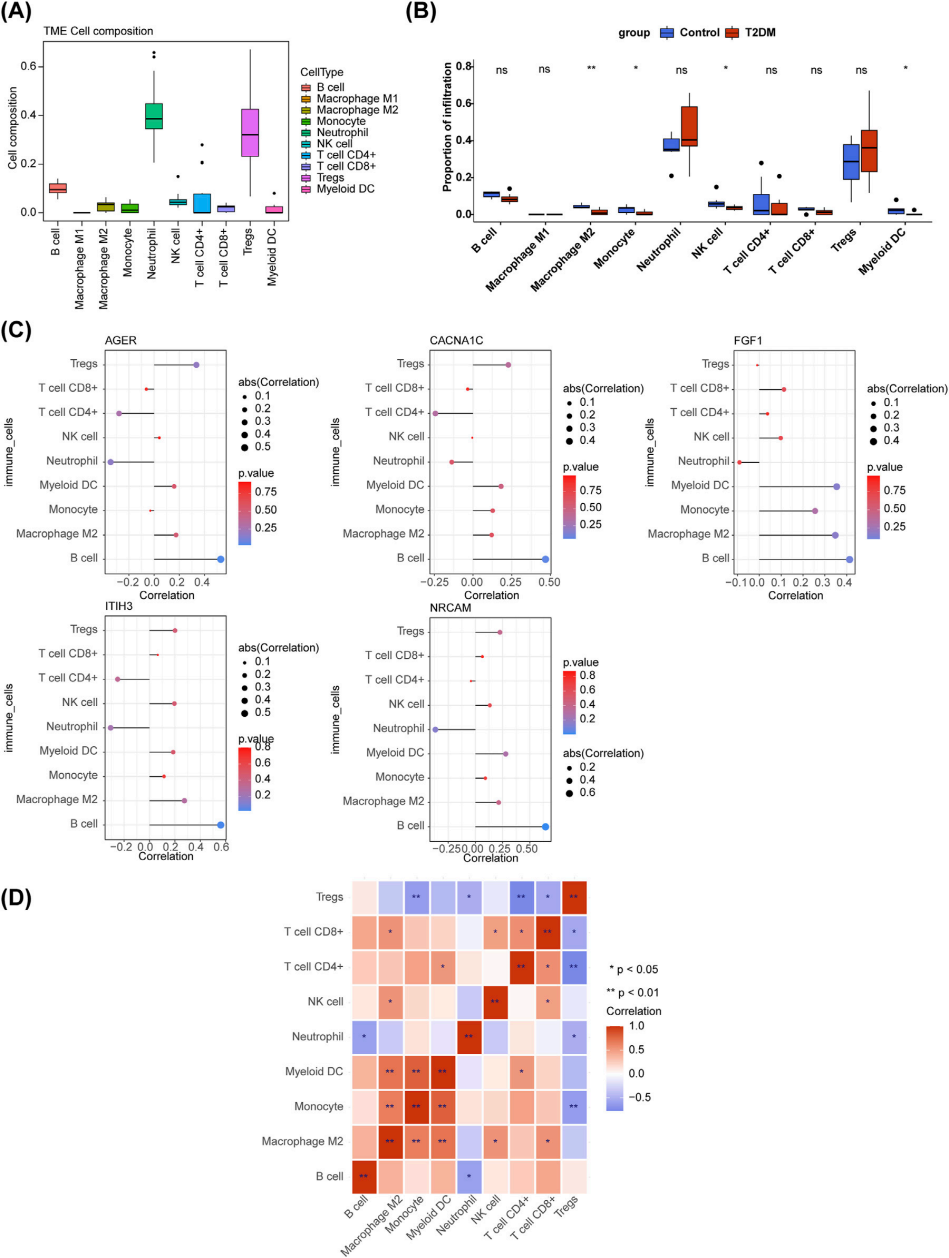
Immune infiltration analysis. **(A)** The boxplot of immune cell composition in all samples. **(B)** Comparison of immune cell infiltration between T2DM and control samples. ns, not significant; *p < 0.05; **p < 0.01. **(C)** Relevance of diagnostic genes and immune cells. **(D)** The heatmap of correaltion analysis among immune cells. *p < 0.05; **p < 0.01.

From the box plot, it could be concluded that the proportion of Neutrophil and Tregs in the samples were relatively high (Figure 5A). M2 Macrophage, Monocyte, Natural killer (NK) cell, and Myeloid dendritic cells (DC) were dramatically different between T2DM and control samples (Figure 5B). The all diagnostic genes had positive and the most significant correlation with B cells (Figure 5C). Correlation analysis showed differences in correlations among 9 immune cells, among them, Monocyte was markedly positively correlated with Myeloid DC, while it was remarkably negatively correlated with Regulatory T cells (Tregs) (Figure 5D).

### 3.6 Construction of gene-drug and TF-miRNA-mRNA networks

**FIGURE 6:**
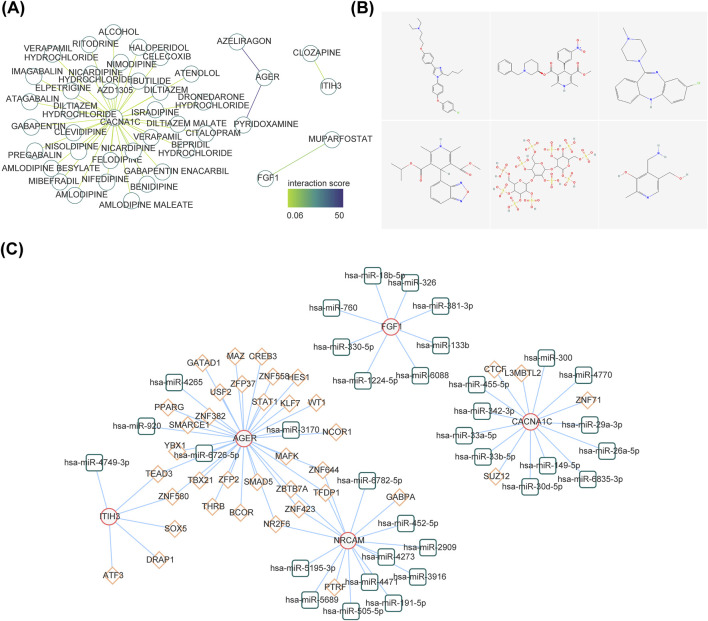
Exploration of potential regulatory mechanisms and drug prediction. **(A)** Construction of the drug-diagnostic gene network. **(B)** The 2D structure of several drugs. **(C)** The interaction among transcription factors, microRNAs (miRNAs), and diagnostic genes. The red line graphic represents diagnostic genes, green represents miRNAs, and orange represents lncRNAs.

The gene-drug network included CACNA1C-Isradipine, CACNA1C-Benidipine, AGER-Pyridoxamine, ITIH3-Clozapine, FGF1-Muparfostat and other relationship pairs (Figure 6A). Figure 6B demonstrated the 2D structure of some drugs. A total of 76 nodes and 44 edges constituted the TF-miRNA-mRNA network, including signal transducer and activator of transcription 1(STAT1)-hsa-miR-3170-AGER, CCCTC-binding factor (CTCF)-hsa-miR-455-5p-CACNA1C, GA binding protein transcription factor Alpha (GABPA) -hsa-miR-452-5p-NRCAM, TEA domain transcription factor 3 (TEAD3)-hsa-miR-4749-3p-ITIH3, ZFP2 zinc finger protein (ZFP2)-hsa-miR-4265-AGER et al. (Figure 6C).

### 3.7 Validation of diagnostic genes expressions by qRT-PCR

**FIGURE 7:**
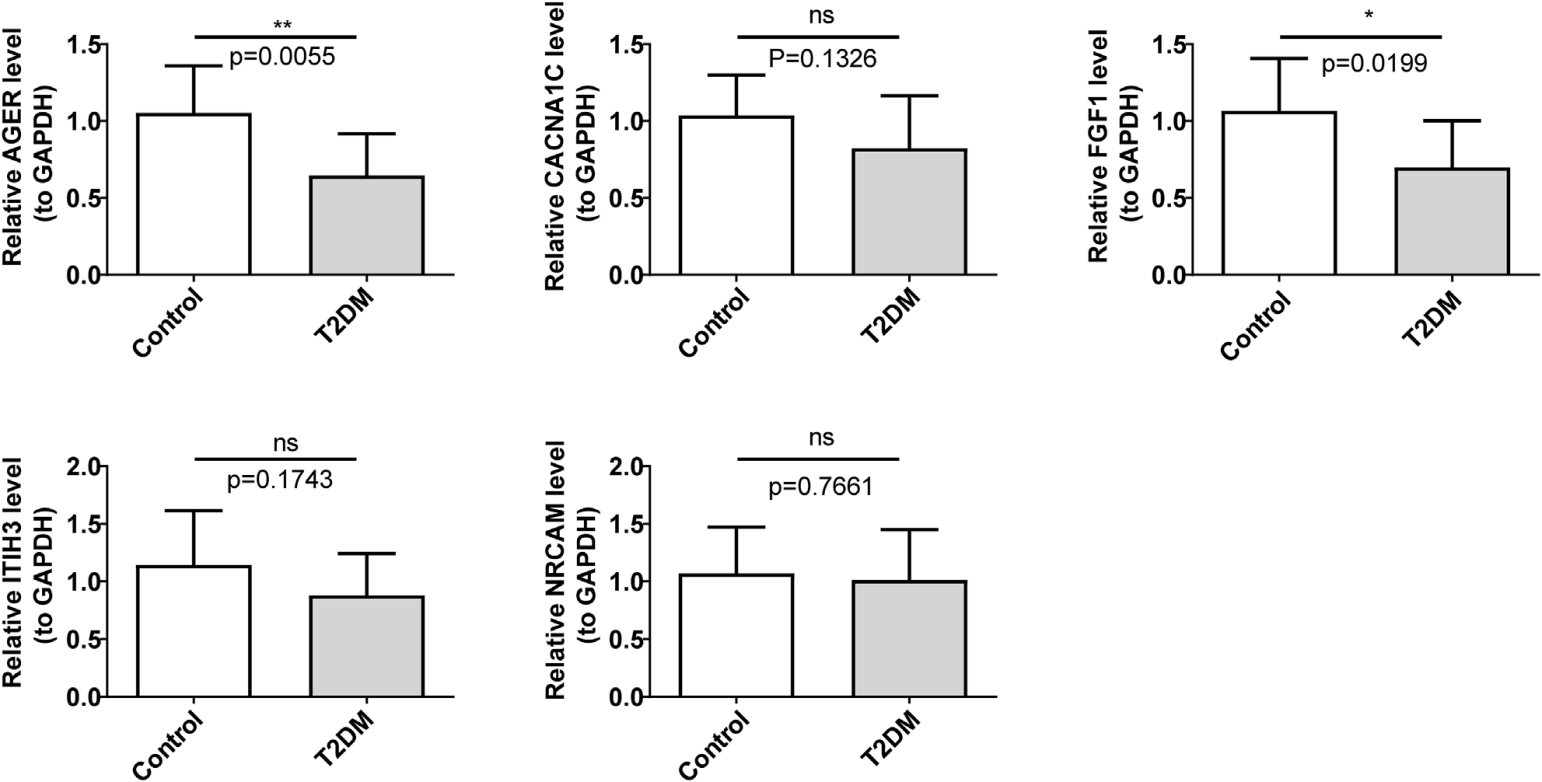
Validation of the expression levels of diagnostic genes. ns, not significant; *p < 0.05; **p < 0.01.

In comparison with the control group, FGF1 and AGER were markedly regulated downwards in the T2DM group (Figure 7). The expression trends of these two diagnostic genes were consistent with the consequences of bioinformatic analysis.

## 4 Discussion

The pathogenesis of T2DM is multifaceted, primarily characterized by insulin resistance and relative insulin insufficiency. The greatest challenge in our treatment of T2DM remains the significant harm caused by its complications. Immune dysfunction is increasingly recognized as a fundamental pathophysiological mechanism underlying both T2DM and its chronic complications (Coope et al., 2016). Studies have displayed that NETs have a crucial role in the initiation and development of diabetes-associated atherosclerosis (AS) (Poznyak et al., 2020), diabetic nephropathy (Zheng et al., 2022), diabetic retinopathy (Magaña-Guerrero et al., 2023) and diabetic foot ulcer (Yang et al., 2023). However, the pathogenesis of NETs and T2DM is still not clear. Through bioinformatics analysis, this study successfully identified and characterized NETs-RGs in T2DM. The diagnostic genes ITIH3, FGF1, NRCAM, AGER, and CACNA1C showed significant association with NETs in T2DM, underscoring their potential as valuable biomarkers for diagnosing T2DM. Furthermore, studies on their mechanisms using immune infiltration analysis, enrichment analysis and regulatory network analysis have provided important insights into the role of NETs-RGs in T2DM. These findings present innovative ideas for the diagnosis and treatment of T2DM patients.

FGF1 is involved in the regulation of glycolipid metabolism. Studies have shown that FGF1 reduces blood sugar by increasing insulin sensitivity, providing a new approach for the treatment of metabolic diseases like fatness, NAFLD and T2DM (Jonker et al., 2012; Suh et al., 2014). It plays a role in coronary heart disease, myocardial ischemia, nerve injury and wound healing (Li, 2019). FGF1 can enhance the healing process of diabetic wounds (Liu Y. et al., 2021). In individuals with diabetes, during the late stages of inflammation in injured tissue, macrophages often remain in an inflammatory state, failing to transition into a reparative phenotype. Consequently, they are unable to secrete factors that facilitate tissue repair, hindering the transition of the wound into the proliferation phase and leading to the development of chronic inflammation (Boniakowski et al., 2018). Our study showed that in contrast with the control group, the proportion of neutrophils in T2DM was remarkably increased, and the proportion of M2 macrophages was significantly reduced, thus promoting the occurrence and development of inflammation. It was also found that excessive neutrophils release NETs in diabetes tissue damage. NETs can activate NLRP 3 inflammasomes in macrophages, thereby releasing IL-1β. These inflammatory cytokines exist in diabetes wounds for much longer than normal, which may prolong the inflammatory phase and inhibit the formation of granulation tissue (Liu et al., 2019). In this study, FGF1 was significantly downregulated in both bioinformatics analysis and qRT-PCR validation in T2DM patients, which may exacerbate abnormal glucose and lipid metabolism and promote the occurrence and development of diabetic complications and delayed wound healing. Therefore, FGF1 may be a latent new aim for the treatment of insulin resistance and T2DM in the future.

AGER is a highly polymorphic gene involved in multiple pathways, such as nuclear factor (NF-κB), protein kinase B (AKT), p38 and mitogen-activated protein (MAP) kinase, which are activated to cause proinflammatory states in the body and may be linked to the growth and development of human autoimmune diseases (Nienhuis et al., 2009), diabetes complications, cancer (Malik et al., 2015), coronary heart disease and lung disease. In this study, we found that intersection genes are trapped in IL-17 signaling pathway, aldosterone synthesis and secretion, cyclic adenosine monophosphate (cAMP), rat sarcoma (RAS), phosphoinositide 3-kinase (PI3K) Akt and mitogen-activated protein kinases (MAPK) signaling pathway, advanced glycation end product (AGE)-receptor for AGE (RAGE) signaling pathway in diabetes complications, which is consistent with literature results. In patients with T2DM, prolonged hyperglycemia leads to the accumulation of advanced glycation end products (AGEs). AGER serves as a receptor for AGEs, initiating AGE-AGER interaction, which in turn activates nuclear factor-kappaB (NF-κB). NF-κB activation triggers the transcription of various pro-inflammatory cytokines such as IL-1α, IL-6, and TNF-α, as well as growth factors and adhesion molecules. This cascade promotes the production of ROS, membrane oxidation, increased membrane permeability, and activation of phospholipase A2 (PLA2). These events collectively contribute to the onset and progression of vascular complications in diabetes (Yuan et al., 2019; Rowisha et al., 2016). Research shows that (Schmidt, 2017), there are significantly more macrophages in diabetes plaques, and the expression of AGER is increased in monocytes/macrophages, the key cells in atherosclerosis, so diabetes plaques show more expression of AGER. This study demonstrated that the expression of AGER in T2DM was lower than that in the control group, which may be associated with the decrease of AGER expression due to the excessive accumulation of AGEs caused by long-term hyperglycemia. Therefore, AGER may get trapped in the occurrence and development of T2DM, but its specific mechanism needs further exploration and research.

CACNA1C encodes the L-type voltage-dependent calcium channel. Insulin secretion is regulated by two distinct phases: early-phase secretion and late-phase secretion, each governed by different regulatory mechanisms. Early-phase insulin secretion is initiated by the uptake of glucose via glucose transporter (GLUT) transporters. Subsequently, the activation of potassium channels leads to the influx of Ca^2+^ through calcium channels, facilitating the release of insulin stored in vesicles through exocytosis (Araki et al., 2019). Glucose-stimulated insulin secretion (GSIS) of pancreatic beta cells plays a key role in maintaining glucose homeostasis and preventing hyperglycemia. It has been shown that Ca^2+^ flows primarily into beta cells via L-type voltage-dependent Ca^2+^ (Ca V) channels, which are necessary for GSIS (Dickerson et al., 2020). This study revealed a significant decrease in the expression of CACNA1C in T2DM patients compared to the control group. This decrease in CACNA1C expression could potentially impair early insulin secretion and GSIS, suggesting it may play a crucial role in the pathogenesis of T2DM. Interestingly, inhibition of excess Ca2+ inflow in pre-diabetic islets by the drug isradipine to prevent Ca2+ induced hypersensitivity may be an option for early intervention, at least to delay the onset of T2DM (Reinbothe et al., 2013; Pĭtre et al., 1999).

In this study, M2 Macrophage, Monocyte, NK cell, Myeloid DC were found to be significantly different between T2DM and control samples. The positive correlation between diagnostic genes and B cells was the strongest and most significant. T2DM is recognized as a chronic low-grade inflammatory condition, driven by the inflammatory activation of both recruited and resident macrophages, known as the classical pathway. This entails the infiltration and activation of macrophages within adipose tissue, which is closely linked to insulin resistance induced by obesity (Wang et al., 2023). Most metabolic organs are made up of dense cells of the inborn and adaptive immune systems. Macrophages play an important role in atherosclerosis (Si et al., 2020). M2 Macrophages has anti-inflammatory and immunomodulatory effects, which helps to promote tissue repair, remodeling, healing, etc. (Li et al., 2022; Rayego-Mateos et al., 2022; Arabpour et al., 2021). This study demonstrated a notable decrease in M2 Macrophages in T2DM patients in comparison with control group, which may be an important reason for delayed wound healing in T2DM. Research has shown that hyperglycemia accelerates the formation of AGEs. Compared with non-diabetes plaques, diabetes plaques have significantly more macrophages. At the same time, diabetes plaques show more expression of AGER (especially in Macrophages). The interaction between AGEs and AGER promotes the occurrence of inflammation and OS, leading to the formation of foam cells in macrophages, which leads to the occurrence and development of diabetes related atherosclerosis (Schmidt, 2017). Therefore, studying the relationship between T2DM and immune cells is particularly important. NK cells are believed to be associated with T2DM by regulating systemic inflammation (Caligiuri, 2008; Zitti and Bryceson, 2018). The study reported that the NK cells contribute to inflammation and insulin resistance induced by obesity (Theurich et al., 2017). The NK cell activity of T2DM patients is significantly reduced and linearly deteriorates with the degree of hyperglycemia, which in turn disrupts the maintenance of the immune system (Dalmas, 2019). This study demonstrated a notable decrease in NK cell levels among T2DM patients, with consistent results across the board. Consequently, comprehending the functional alterations of NK cells presents novel avenues for addressing T2DM resulting from obesity.

This study was conducted through DrugBank (https://go.drugbank.com/) online databases predict targeted drugs related to diagnostic genes. In our study, we found that Pyridoxamine was one of the drugs searched for by AGER. Pyridoxamine is a derivative of vitamin B6, an inhibitor of advanced glycation and lipid oxidation end products. Studies have shown that pyridoxamine can inhibit the kidney lesions caused by AGEs, and can also significantly reduce plasma cholesterol, triglyceride, creatinine and urinary albumin in diabetic rats, and reduce the content of skin collagen AGEs (Khalifah et al., 1999). Muparfostat is also known as PI-88, an inhibitor of heparinase (HPSE). HPSE is an endo-β-D-glucuronidase capable of degrading heparan sulfate (HS). Studies have reported that the increase of blood glucose can lead to the upregulation of HPSE expression, and with the prolongation of diabetes, HPSE may cause more serious damage to islet function (Simeonovic et al., 2013; Dhounchak et al., 2021). At the same time, HS acts in the FGFs signaling pathway. FGFRS mediates FGFs signal transduction through heparan sulfate or Klotho-dependent ways. Long-term hyperglycemia leads to the overexpression of HPSE and the destruction of HS, which in turn affects the FGFs signaling pathway and has a negative impact on blood glucose control (Masola et al., 2011; Ferro et al., 2004). In the future, specific studies can be conducted on the treatment of T2DM with Pyridoxamine and Muparfostat.

In this study, the relationship between T2DM and NETs-RGs was discussed through bioinformatics analysis, and five diagnostic genes related to NETs were identified in T2DM, including ITIH3, FGF1, NRCAM, AGER, and CACNA1C. The mechanism of action of diagnostic genes on T2DM was also explored from different perspectives. It provides a new way to study the diagnosis and treatment of T2DM. However, the method of confidence generation limited our ability to predict the TFs and related regulatory networks of only five diagnostic genes using the online database. The association between certain diagnostic genes, such as ITIH3 and NRCAM, and diabetes remains understudied. Only FGF1 and AGER genes were validated by qRT PCR in this study, while the other three genes were not validated. This may be related to small sample sizes, limited to specific populations, or possible biases during the validation process. As a result, the other three were not validated successfully. The precise regulatory mechanisms of NETs-RGs AGER, FGF1, and CACNA1C in T2DM have not been fully elucidated. Moving forward, we will continue to closely monitor the research progress concerning these diagnostic genes and their roles in T2DM.

## Data Availability

The datasets presented in this study can be found in online repositories. The names of the repository/repositories and accession number(s) can be found in the article/Supplementary Material.

## References

[B1] Amorim M. Martins B. Fernandes R. (2023). Immune fingerprint in diabetes: ocular surface and retinal inflammation. Int. J. Mol. Sci. 24, 9821. 10.3390/ijms24129821 37372968 PMC10298084

[B2] Arabpour M. Saghazadeh A. Rezaei N. (2021). Anti-inflammatory and M2 macrophage polarization-promoting effect of mesenchymal stem cell-derived exosomes. Int. Immunopharmacol. 97, 107823. 10.1016/j.intimp.2021.107823 34102486

[B3] Araki K. Araki A. Honda D. Izumoto T. Hashizume A. Hijikata Y. (2019). TDP-43 regulates early-phase insulin secretion via CaV1.2-mediated exocytosis in islets. J. Clin. Investig. 129, 3578–3593. 10.1172/jci124481 31355778 PMC6715357

[B4] Berbudi A. Rahmadika N. Tjahjadi A. I. Ruslami R. (2020). Type 2 diabetes and its impact on the immune system. Curr. Diabetes Rev. 16, 442–449. 10.2174/1573399815666191024085838 31657690 PMC7475801

[B5] Boniakowski A. E. Kimball A. S. Joshi A. Schaller M. Davis F. M. denDekker A. (2018). Murine macrophage chemokine receptor CCR2 plays a crucial role in macrophage recruitment and regulated inflammation in wound healing. Eur. J. Immunol. 48, 1445–1455. 10.1002/eji.201747400 29879295 PMC6371802

[B6] Borissoff J. I. Joosen I. A. Versteylen M. O. Brill A. Fuchs T. A. Savchenko A. S. (2013). Elevated levels of circulating DNA and chromatin are independently associated with severe coronary atherosclerosis and a prothrombotic state. Arterioscler. Thromb. Vasc. Biol. 33, 2032–2040. 10.1161/atvbaha.113.301627 23818485 PMC3806482

[B7] Brinkmann V. Zychlinsky A. (2007). Beneficial suicide: why neutrophils die to make NETs. Nat. Rev. Microbiol. 5, 577–582. 10.1038/nrmicro1710 17632569

[B8] Bruschi M. Bonanni A. Petretto A. Vaglio A. Pratesi F. Santucci L. (2020). Neutrophil extracellular traps profiles in patients with incident systemic lupus erythematosus and lupus nephritis. J. Rheumatol. 47, 377–386. 10.3899/jrheum.181232 31092713 PMC6917988

[B9] Caligiuri M. A. (2008). Human natural killer cells. Blood 112, 461–469. 10.1182/blood-2007-09-077438 18650461 PMC2481557

[B10] Caveolin S. A. (2020). Caveolin: a new link between diabetes and ad. Cell. Mol. Neurobiol. 40 (7), 1059–1066. Epub 2020 Jan 23. PMID: 31974905. 10.1007/s10571-020-00796-4 31974905 PMC11448860

[B11] Coope A. Torsoni A. S. Velloso L. A. (2016). Mechanisms in endocrinology: metabolic and inflammatory pathways on the pathogenesis of type 2 diabetes. Eur. J. Endocrinol. 174, R175–R187. 10.1530/eje-15-1065 26646937

[B12] Dalmas E. (2019). Role of innate immune cells in metabolism: from physiology to type 2 diabetes. Semin. Immunopathol. 41, 531–545. 10.1007/s00281-019-00736-5 30953161

[B13] Dhounchak S. Popp S. K. Brown D. J. Laybutt D. R. Biden T. J. Bornstein S. R. (2021). Heparan sulfate proteoglycans in beta cells provide a critical link between endoplasmic reticulum stress, oxidative stress and type 2 diabetes. PLoS One 16, e0252607. 10.1371/journal.pone.0252607 34086738 PMC8177513

[B14] Dickerson M. T. Dadi P. K. Butterworth R. B. Nakhe A. Y. Graff S. M. Zaborska K. E. (2020). Tetraspanin-7 regulation of L-type voltage-dependent calcium channels controls pancreatic β-cell insulin secretion. J. Physiol. 598, 4887–4905. 10.1113/jp279941 32790176 PMC8095317

[B15] Ferro V. Hammond E. Fairweather J. K. (2004). The development of inhibitors of heparanase, a key enzyme involved in tumour metastasis, angiogenesis and inflammation. Mini Rev. Med. Chem. 4, 693–702. 10.2174/1389557043403729 15279603

[B16] Hawez A. Al-Haidari A. Madhi R. Rahman M. Thorlacius H. (2019). MiR-155 regulates PAD4-dependent formation of neutrophil extracellular traps. Front. Immunol. 10, 2462. 10.3389/fimmu.2019.02462 31736940 PMC6838784

[B17] Hirota T. Levy J. H. Iba T. (2020). The influence of hyperglycemia on neutrophil extracellular trap formation and endothelial glycocalyx damage in a mouse model of type 2 diabetes. Microcirculation 27, e12617. 10.1111/micc.12617 32125048

[B18] Itariu B. K. Stulnig T. M. (2014). Autoimmune aspects of type 2 diabetes mellitus - a mini-review. Gerontology 60, 189–196. 10.1159/000356747 24457898

[B19] Jo S. Fang S. (2021). Therapeutic strategies for diabetes: immune modulation in pancreatic β cells. Front. Endocrinol. (Lausanne) 12, 716692. 10.3389/fendo.2021.716692 34484126 PMC8415970

[B20] Jonker J. W. Suh J. M. Atkins A. R. Ahmadian M. Li P. Whyte J. (2012). A PPARγ-FGF1 axis is required for adaptive adipose remodelling and metabolic homeostasis. Nature 485, 391–394. 10.1038/nature10998 22522926 PMC3358516

[B21] Khalifah R. G. Baynes J. W. Hudson B. G. (1999). Amadorins: novel post-Amadori inhibitors of advanced glycation reactions. Biochem. Biophys. Res. Commun. 257, 251–258. 10.1006/bbrc.1999.0371 10198198

[B22] Langfelder P. Horvath S. (2008). WGCNA: an R package for weighted correlation network analysis. BMC Bioinforma. 9, 559. 10.1186/1471-2105-9-559 PMC2631488 19114008

[B23] Lewis H. D. Liddle J. Coote J. E. Atkinson S. J. Barker M. D. Bax B. D. (2015). Inhibition of PAD4 activity is sufficient to disrupt mouse and human NET formation. Nat. Chem. Biol. 11, 189–191. 10.1038/nchembio.1735 25622091 PMC4397581

[B24] Li H. D. You Y. K. Shao B. Y. Wu W. F. Wang Y. F. Guo J. B. (2022). Roles and crosstalks of macrophages in diabetic nephropathy. Front. Immunol. 13, 1015142. 10.3389/fimmu.2022.1015142 36405700 PMC9666695

[B25] Li X. (2019). The FGF metabolic axis. Front. Med. 13, 511–530. 10.1007/s11684-019-0711-y 31495905 PMC7102389

[B26] Liu D. Yang P. Gao M. Yu T. Shi Y. Zhang M. (2019). NLRP3 activation induced by neutrophil extracellular traps sustains inflammatory response in the diabetic wound. Clin. Sci. (Lond). 133, 565–582. 10.1042/cs20180600 30626731

[B27] Liu T. T. Li R. Huo C. Li J. P. Yao J. Ji X. L. (2021a). Identification of CDK2-related immune forecast model and ceRNA in lung adenocarcinoma, a pan-cancer analysis. Front. Cell. Dev. Biol. 9, 682002. 10.3389/fcell.2021.682002 34409029 PMC8366777

[B28] Liu Y. Liu Y. Deng J. Li W. Nie X. (2021b). Fibroblast growth factor in diabetic foot ulcer: progress and therapeutic prospects. Front. Endocrinol. (Lausanne) 12, 744868. 10.3389/fendo.2021.744868 34721299 PMC8551859

[B29] Lowe W. L. Jr. Boyd F. T. Clarke D. W. Raizada M. K. Hart C. LeRoith D. (1986). Development of brain insulin receptors: structural and functional studies of insulin receptors from whole brain and primary cell cultures. Endocrinology 119, 25–35. 10.1210/endo-119-1-25 3522210

[B30] Magaña-Guerrero F. S. Aguayo-Flores J. E. Buentello-Volante B. Zarco-Ávila K. Sánchez-Cisneros P. Castro-Salas I. (2023). Spontaneous neutrophil extracellular traps release are inflammatory markers associated with hyperglycemia and renal failure on diabetic retinopathy. Biomedicines 11, 1791. 10.3390/biomedicines11071791 37509431 PMC10376331

[B31] Malik P. Chaudhry N. Mittal R. Mukherjee T. K. (2015). Role of receptor for advanced glycation end products in the complication and progression of various types of cancers. Biochim. Biophys. Acta 1850, 1898–1904. 10.1016/j.bbagen.2015.05.020 26028296

[B32] Marassi M. Fadini G. P. (2023). The cardio-renal-metabolic connection: a review of the evidence. Cardiovasc Diabetol. 22 (1), 195. PMID: 37525273; PMCID: PMC10391899. 10.1186/s12933-023-01937-x 37525273 PMC10391899

[B33] Masola V. Gambaro G. Tibaldi E. Onisto M. Abaterusso C. Lupo A. (2011). Regulation of heparanase by albumin and advanced glycation end products in proximal tubular cells. Biochim. Biophys. Acta 1813, 1475–1482. 10.1016/j.bbamcr.2011.05.004 21600934

[B34] Nienhuis H. L. Westra J. Smit A. J. Limburg P. C. Kallenberg C. G. Bijl M. (2009). AGE and their receptor RAGE in systemic autoimmune diseases: an inflammation propagating factor contributing to accelerated atherosclerosis. Autoimmunity 42, 302–304. 10.1080/08916930902831746 19811283

[B35] Papayannopoulos V. Metzler K. D. Hakkim A. Zychlinsky A. (2010). Neutrophil elastase and myeloperoxidase regulate the formation of neutrophil extracellular traps. J. Cell. Biol. 191, 677–691. 10.1083/jcb.201006052 20974816 PMC3003309

[B36] Parker H. Winterbourn C. C. (2012). Reactive oxidants and myeloperoxidase and their involvement in neutrophil extracellular traps. Front. Immunol. 3, 424. 10.3389/fimmu.2012.00424 23346086 PMC3549523

[B37] Petretto A. Bruschi M. Pratesi F. Croia C. Candiano G. Ghiggeri G. (2019). Neutrophil extracellular traps (NET) induced by different stimuli: a comparative proteomic analysis. PLoS One 14, e0218946. 10.1371/journal.pone.0218946 31283757 PMC6613696

[B38] Pĭtre M. Gaudreault N. Santuré M. Nadeau A. Bachelard H. (1999). Isradipine and insulin sensitivity in hypertensive rats. Am. J. Physiol. 276, E1038–E1048. 10.1152/ajpendo.1999.276.6.E1038 10362616

[B39] Poznyak A. Grechko A. V. Poggio P. Myasoedova V. A. Alfieri V. Orekhov A. N. (2020). The diabetes mellitus-atherosclerosis connection: the role of lipid and glucose metabolism and chronic inflammation. Int. J. Mol. Sci. 21, 1835. 10.3390/ijms21051835 32155866 PMC7084712

[B40] Rayego-Mateos S. Marquez-Expósito L. Rodrigues-Diez R. Sanz A. B. Guiteras R. Doladé N. (2022). Molecular mechanisms of kidney injury and repair. Int. J. Mol. Sci. 23, 1542. 10.3390/ijms23031542 35163470 PMC8835923

[B41] Reeves E. P. Lu H. Jacobs H. L. Messina C. G. Bolsover S. Gabella G. (2002). Killing activity of neutrophils is mediated through activation of proteases by K+ flux. Nature 416, 291–297. 10.1038/416291a 11907569

[B42] Reinbothe T. M. Alkayyali S. Ahlqvist E. Tuomi T. Isomaa B. Lyssenko V. (2013). The human L-type calcium channel Cav1.3 regulates insulin release and polymorphisms in CACNA1D associate with type 2 diabetes. Diabetologia 56, 340–349. 10.1007/s00125-012-2758-z 23229155

[B43] Robin X. Turck N. Hainard A. Tiberti N. Lisacek F. Sanchez J. C. (2011). pROC: an open-source package for R and S+ to analyze and compare ROC curves. BMC Bioinforma. 12, 77. 10.1186/1471-2105-12-77 PMC3068975 21414208

[B44] Rowisha M. El-Batch M. El Shikh T. El Melegy S. Aly H. (2016). Soluble receptor and gene polymorphism for AGE: relationship with obesity and cardiovascular risks. Pediatr. Res. 80, 67–71. 10.1038/pr.2016.55 26991258

[B45] Schmidt A. M. (2017). 22016 ATVB plenary lecture: receptor for advanced glycation endproducts and implications for the pathogenesis and treatment of cardiometabolic disorders: spotlight on the macrophage. Arterioscler. Thromb. Vasc. Biol. 37, 613–621. 10.1161/atvbaha.117.307263 28183700 PMC5364055

[B46] Si Y. Liu J. Shan W. Zhang Y. Han C. Wang R. (2020). Association of lymphocyte-to-monocyte ratio with total coronary plaque burden in patients with coronary artery disease. Coron. Artery Dis. 31, 650–655. 10.1097/mca.0000000000000857 32097130 PMC7531493

[B47] Simeonovic C. J. Ziolkowski A. F. Wu Z. Choong F. J. Freeman C. Parish C. R. (2013). Heparanase and autoimmune diabetes. Front. Immunol. 4, 471. 10.3389/fimmu.2013.00471 24421779 PMC3872651

[B48] Smith C. K. Vivekanandan-Giri A. Tang C. Knight J. S. Mathew A. Padilla R. L. (2014). Neutrophil extracellular trap-derived enzymes oxidize high-density lipoprotein: an additional proatherogenic mechanism in systemic lupus erythematosus. Arthritis Rheumatol. 66, 2532–2544. 10.1002/art.38703 24838349 PMC4146708

[B49] Su R. Jin C. Bu H. Xiang J. Zhou L. Jin C. (2022). Development and validation of an immune-related prognostic signature in cervical cancer. Front. Oncol. 12, 861392. 10.3389/fonc.2022.861392 35651784 PMC9148954

[B50] Suh J. M. Jonker J. W. Ahmadian M. Goetz R. Lackey D. Osborn O. (2014). Endocrinization of FGF1 produces a neomorphic and potent insulin sensitizer. Nature 513, 436–439. 10.1038/nature13540 25043058 PMC4184286

[B67] Surguchov A. C. (2020). A new link between diabetes and AD. Cell. Mol. Neurobiol. 40 (7), 1059–1066. 10.1007/s10571-020-00796-4 31974905 PMC11448860

[B51] Tan T. E. Wong T. Y. (2023). Diabetic retinopathy: looking forward to 2030. Front. Endocrinol. (Lausanne) 13, 1077669. PMID: 36699020; PMCID: PMC9868457. 10.3389/fendo.2022.1077669 36699020 PMC9868457

[B52] Theurich S. Tsaousidou E. Hanssen R. Lempradl A. M. Mauer J. Timper K. (2017). IL-6/Stat3-Dependent induction of a distinct, obesity-associated NK cell subpopulation deteriorates energy and glucose homeostasis. Cell. Metab. 26, 171–184. 10.1016/j.cmet.2017.05.018 28683285

[B53] Thipsawat S. (2021). Early detection of diabetic nephropathy in patient with type 2 diabetes mellitus: a review of the literature. Diab Vasc. Dis. Res. 18 (6), 14791641211058856. 10.1177/14791641211058856 34791910 PMC8606936

[B54] Wang H. W. Tang J. Sun L. Li Z. Deng M. Dai Z. (2023). Mechanism of immune attack in the progression of obesity-related type 2 diabetes. World J. Diabetes 14, 494–511. 10.4239/wjd.v14.i5.494 37273249 PMC10236992

[B55] Wang Y. Li M. Stadler S. Correll S. Li P. Wang D. (2009). Histone hypercitrullination mediates chromatin decondensation and neutrophil extracellular trap formation. J. Cell. Biol. 184, 205–213. 10.1083/jcb.200806072 19153223 PMC2654299

[B56] Wang Y. Wang Z. Sun J. Qian Y. (2021). Identification of HCC subtypes with different prognosis and metabolic patterns based on mitophagy. Front. Cell. Dev. Biol. 9, 799507. 10.3389/fcell.2021.799507 34977039 PMC8716756

[B57] Wu J. Zhang F. Zheng X. Zhang J. Cao P. Sun Z. (2022). Identification of renal ischemia reperfusion injury subtypes and predictive strategies for delayed graft function and graft survival based on neutrophil extracellular trap-related genes. Front. Immunol. 13, 1047367. 10.3389/fimmu.2022.1047367 36532016 PMC9752097

[B58] Xu H. Jiang J. Chen W. Li W. Chen Z. (2019). Vascular macrophages in atherosclerosis. J. Immunol. Res. 2019, 4354786. 10.1155/2019/4354786 31886303 PMC6914912

[B59] Yang L. Liu Q. Zhang X. Liu X. Zhou B. Chen J. (2020). DNA of neutrophil extracellular traps promotes cancer metastasis via CCDC25. Nature 583, 133–138. 10.1038/s41586-020-2394-6 32528174

[B60] Yang S. Wang S. Chen L. Wang Z. Chen J. Ni Q. (2023). Neutrophil extracellular traps delay diabetic wound healing by inducing endothelial-to-mesenchymal transition via the hippo pathway. Int. J. Biol. Sci. 19 (1), 347–361. 10.7150/ijbs.78046 36594092 PMC9760440

[B61] Yu G. Wang L. G. Han Y. He Q. Y. (2012). clusterProfiler: an R package for comparing biological themes among gene clusters. Omics 16, 284–287. 10.1089/omi.2011.0118 22455463 PMC3339379

[B66] Yang K. Wang Y. Li Y. W. Chen Y. G. Xing N. Lin H. B. (2022). Progress in the treatment of diabetic peripheral neuropathy. Biomed Pharmacother. 148, 112717. 10.1016/j.biopha.2022.112717 35193039

[B62] Yuan T. Yang T. Chen H. Fu D. Hu Y. Wang J. (2019). New insights into oxidative stress and inflammation during diabetes mellitus-accelerated atherosclerosis. Redox Biol. 20, 247–260. 10.1016/j.redox.2018.09.025 30384259 PMC6205410

[B63] Zheng F. Ma L. Li X. Wang Z. Gao R. Peng C. (2022). Neutrophil extracellular traps induce glomerular endothelial cell dysfunction and pyroptosis in diabetic kidney disease. Diabetes 71, 2739–2750. 10.2337/db22-0153 36095260

[B64] Zhou T. Hu Z. Yang S. Sun L. Yu Z. Wang G. (2018). Role of adaptive and innate immunity in type 2 diabetes mellitus. J. Diabetes Res. 2018, 7457269. 10.1155/2018/7457269 30533447 PMC6250017

[B65] Zitti B. Bryceson Y. T. (2018). Natural killer cells in inflammation and autoimmunity. Cytokine Growth Factor Rev. 42, 37–46. 10.1016/j.cytogfr.2018.08.001 30122459

