# Supplementary figures and images for: Identification and validation of diagnostic genes associated with neutrophil extracellular traps of type 2 diabetes mellitus
Source: Front Genet. 2024 Sep 4;15:1373807. doi: 10.3389/fgene.2024.1373807 (PMC11408200; doi:10.3389/fgene.2024.1373807)

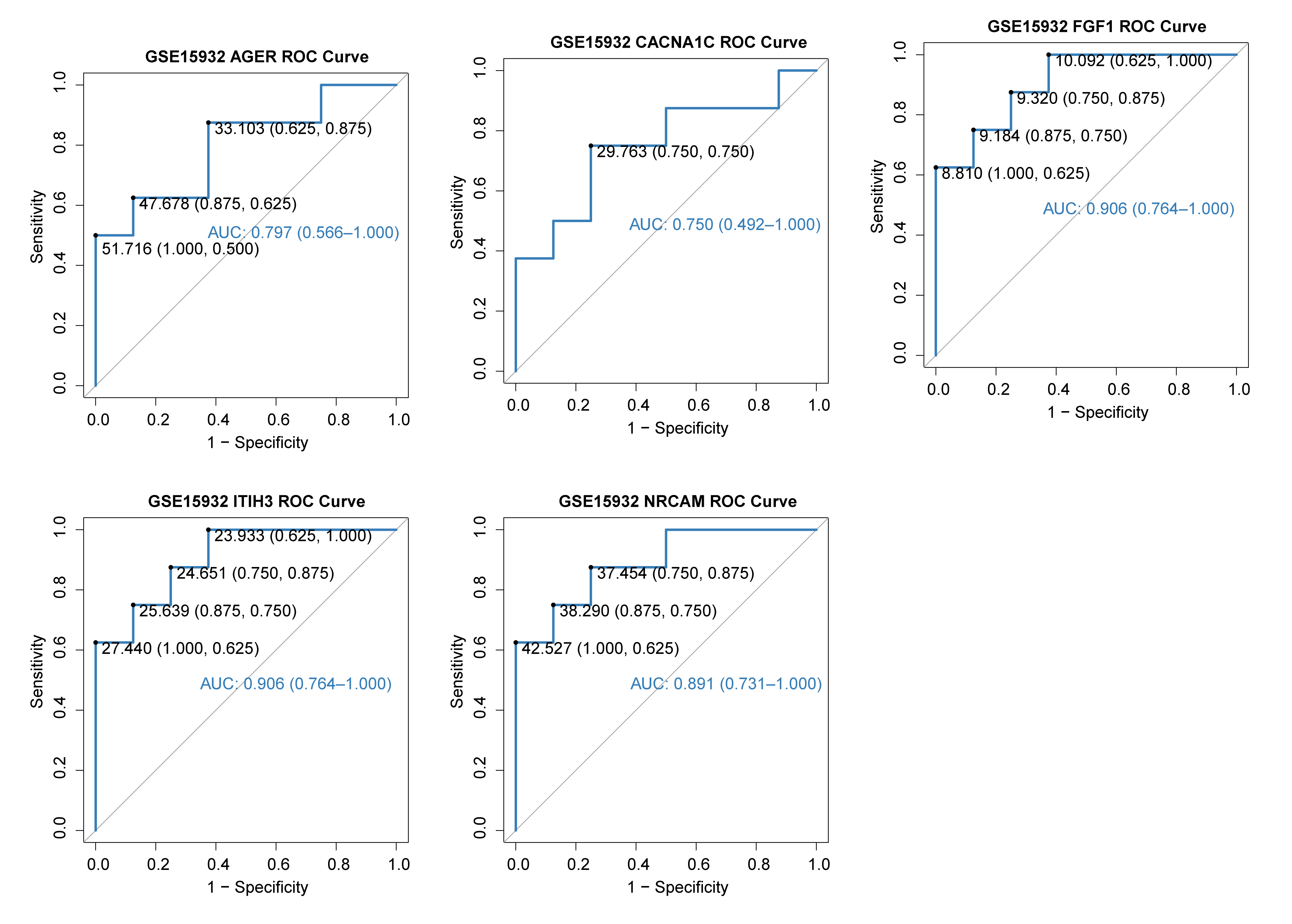

Supplement: Supplementary file 2 [file Image3.TIF]

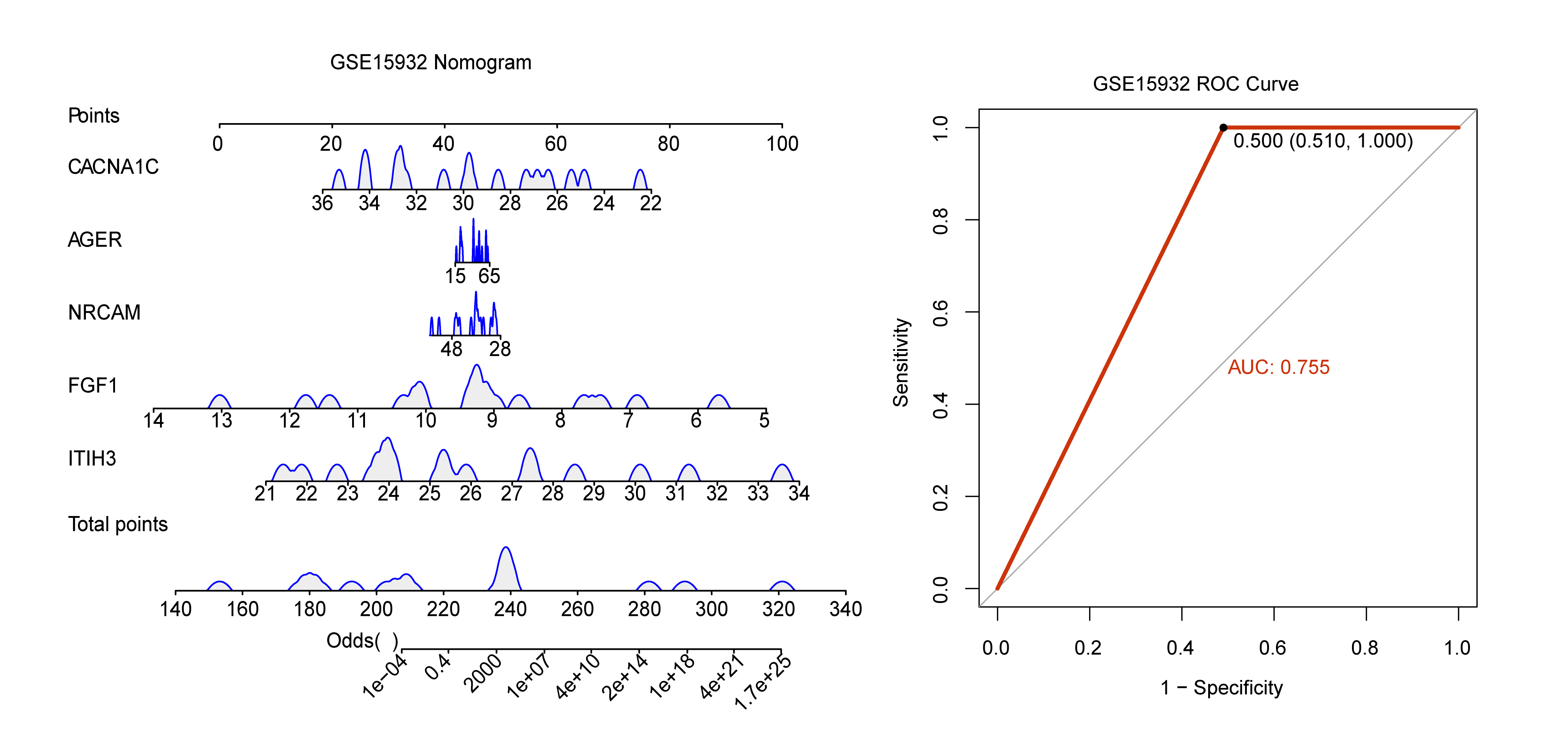

Supplement: Supplementary file 3 [file Image4.TIF]

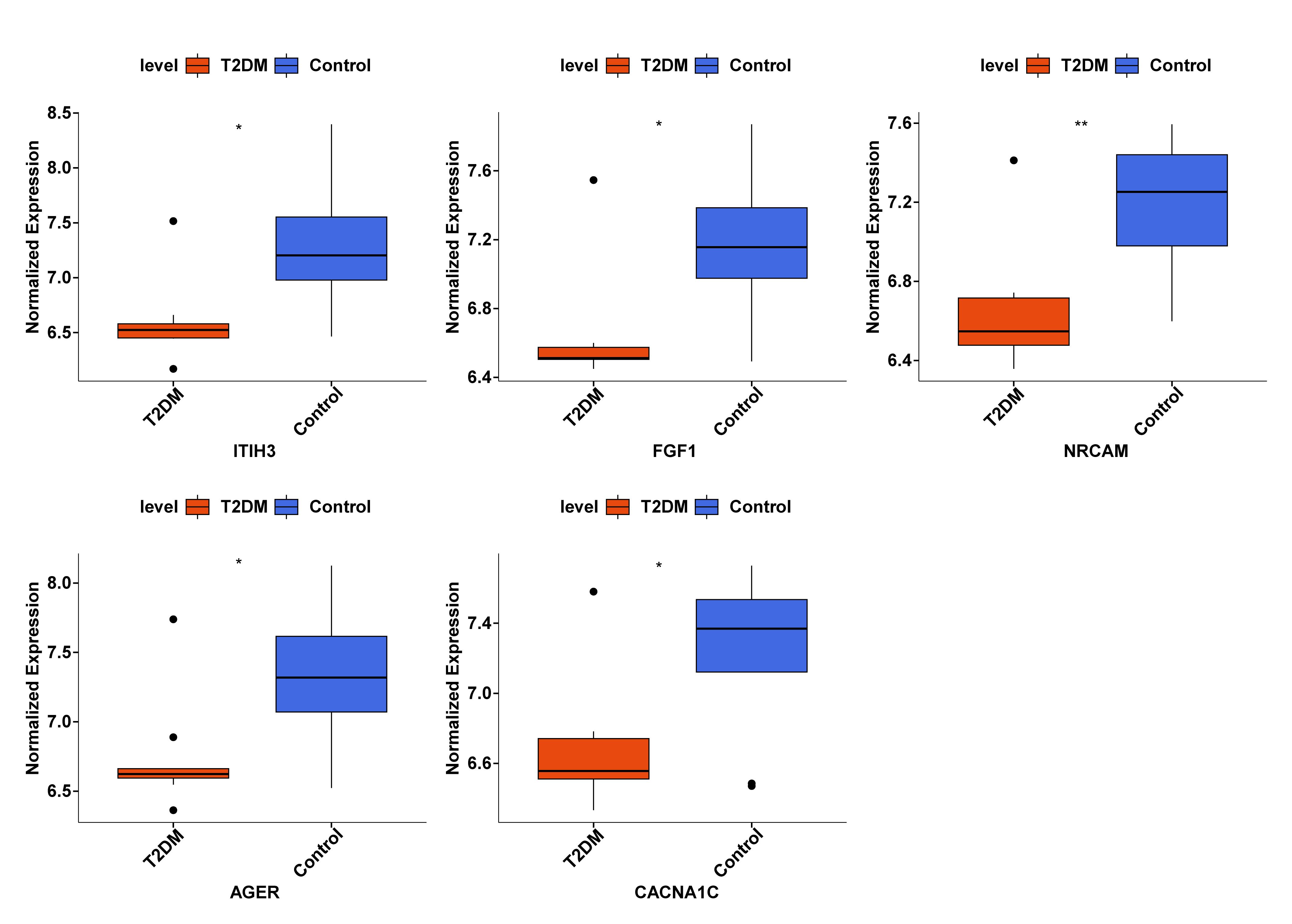

Supplement: Supplementary file 4 [file Image2.TIF]

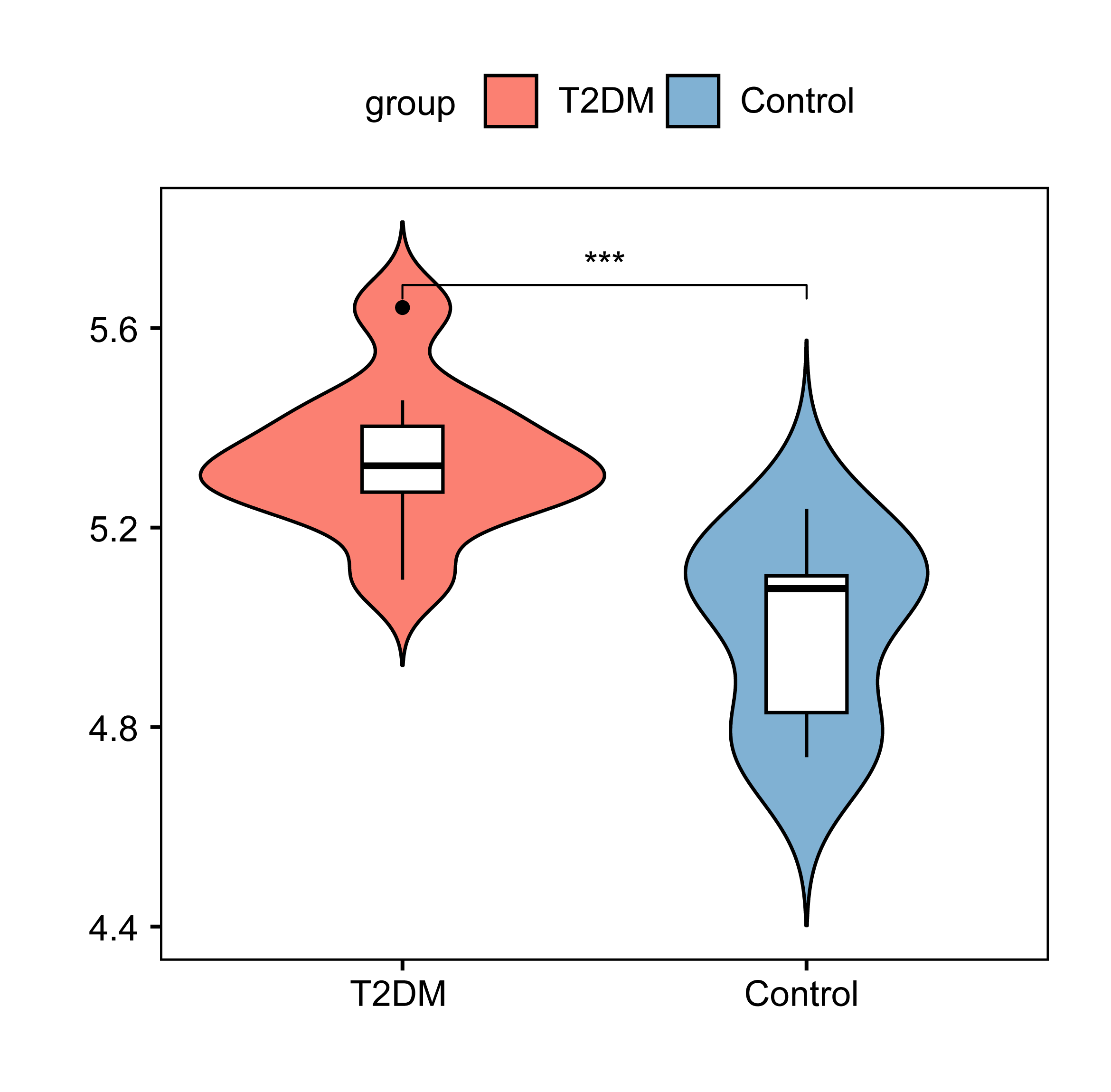

Supplement: Supplementary file 5 [file Image1.TIF]
